# Vitamin D Improves Cognitive Impairment and Alleviates Ferroptosis via the Nrf2 Signaling Pathway in Aging Mice

**DOI:** 10.3390/ijms242015315

**Published:** 2023-10-18

**Authors:** Jiaxin Li, Yang Cao, Jie Xu, Jing Li, Chunmei Lv, Qiang Gao, Chi Zhang, Chongfei Jin, Ran Wang, Runsheng Jiao, Hui Zhu

**Affiliations:** College of Basic Medical Sciences, Heilongjiang Academy of Medical Sciences, Harbin Medical University, Harbin 150081, China; lijiaxin0824@163.com (J.L.); caoyanghmu@163.com (Y.C.); xujie@ems.hrbmu.edu.cn (J.X.); jing070822@163.com (J.L.); angel200202003@163.com (C.L.); gaoqiangphysiology@hrbmu.edu.cn (Q.G.); zhangchihmu@163.com (C.Z.); 18893465363@163.com (C.J.); wangranroadlight@163.com (R.W.); jiaors@ems.hrbmu.edu.cn (R.J.)

**Keywords:** neurodegenerative diseases, aging, ferroptosis, vitamin D

## Abstract

Ferroptosis is an iron-dependent mode of cell death associated with the occurrence and development of age-related neurodegenerative diseases. Currently, there are no effective drugs available to prevent or treat these aging-related neurodegenerative diseases. Vitamin D (VD) is an antioxidant and immunomodulator, but its relationship with ferroptosis in aging-related neurodegenerative diseases has not been extensively studied. In this study, we aimed to investigate the role of VD in learning and memory in aging mice. To examine whether VD protects aging hippocampal neurons, we used physiologically active 1,25(OH)_2_D_3_. We established aging models in vivo (C57BL/6 mice) and in vitro (HT22 cells) using D-galactose (D-gal). The results demonstrated that VD could improve learning and memory in mice aged via the use of D-gal, and it reduced damage to hippocampal neurons. VD could regulate ferroptosis-related proteins (increasing GPX4 expression and decreasing ACSL4 and ALOX15 protein expression levels), increasing GSH levels, reducing MDA and intracellular and mitochondrial ROS levels, as well as total iron and Fe^2+^ levels, and improving mitochondrial morphology, thereby alleviating ferroptosis in aging hippocampal neurons. Additionally, VD activated the VDR/Nrf2/HO-1 signaling pathway, thereby inhibiting ferroptosis. Notably, when the VDR was knocked down, VD lost its ability to activate Nrf2. Consequently, inhibiting Nrf2 decreased the protective effect of VD against ferroptosis in aged hippocampal neurons. In summary, VD activates the Nrf2/HO-1 signaling pathway through the VDR, effectively preventing ferroptosis induced by aging in hippocampal neurons.

## 1. Introduction

Aging refers to the gradual degradation and loss of physiological functions over time, resulting in an increased risk of cardiovascular, neurodegenerative, metabolic, and immune diseases [1,2,3]. One of the main causes of the reduction in brain function is aging. Neurodegenerative disorders associated with aging have significant impacts on the quality of life of older individuals. Research suggests that these diseases affect up to 50 million people worldwide, and approximately 10 million new cases are reported each year (https://www.who.int/news-room/fact-sheets/detail/dementia, accessed on 15 March 2023). Therefore, it is crucial to find effective treatments to ameliorate aging-related neurodegenerative diseases.

The metabolism of D-galactose (D-gal) leads to the production of superoxide anions, which directly damage tissues and organs. This process accelerates neurodegeneration, ultimately resulting in neurodegenerative symptoms. The prolonged administration of high doses of D-gal can induce brain aging similar to the natural aging process in humans, leading to memory impairment, mitochondrial dysfunction, neuronal degeneration, and enhanced oxidative stress [4,5,6]. The D-gal-induced aging model is widely used to simulate aging in the brain and is extensively used for researching anti-aging treatments. Notably, iron levels in tissues increase with age [7], rendering the tissue more susceptible to ferroptosis.

The concept of ferroptosis was introduced by Dixon et al. in 2012 [8]. Ferroptosis, which is an iron-dependent form of cell death, is characterized by reduced mitochondrial volume, increased membrane density, and decreased or absent cristae. The key features of ferroptosis include the accumulation of reactive oxygen species (ROS), the depletion of glutathione (GSH), and decreased activity of glutathione peroxidase 4 (GPX4). Ferroptosis is involved in the development of multiple neurodegenerative diseases, including Parkinson’s disease, Huntington’s disease, Alzheimer’s disease, and amyotrophic lateral sclerosis [9,10,11,12]. Hambright et al. found that Gpx4BIKO mice exhibited significant deficits in spatial learning, memory function, and hippocampal neurodegeneration compared to control mice. Treatment with a small-molecule ferroptosis inhibitor ameliorated neurodegeneration in these mice [13]. Additionally, evidence suggests that loss of the iron exporter protein ferroportin 1 (Fpn 1) participates in neuronal loss and memory impairment in AD, and targeting Fpn 1 or inhibiting ferroptosis could be a promising therapeutic strategy for AD [14]. In addition, ferroptosis is involved in oxygen–glucose deprivation/reperfusion (OGD/R)-induced neuronal damage, and kaempferol inhibits OGD/R-induced ferroptosis by activating the nuclear factor-erythroid 2-related factor 2 (Nrf2)/recombinant solute carrier family 7, member 11 (SLC7A11)/GPX4 pathway [15]. Therefore, inhibiting ferroptosis may be a valuable strategy for preventing age-related neurodegenerative diseases.

Vitamin D (VD) plays a vital role in bone calcium synthesis, neurotrophic regulation, immune regulation, and brain plasticity [16,17,18]. The most active VD metabolite is 1,25(OH)_2_D_3_, which exerts biological effects by binding to the vitamin D receptor (VDR) [19]. Studies on the impact of VD on aging have primarily focused on metabolic diseases, kidney diseases, and cardiovascular diseases [20,21,22], and there have been few studies on its regulatory effects on the nervous system. Some studies have demonstrated that VD’s activation of the VDR inhibits osteoblast ferroptosis by enhancing the Nrf2/GPX4 signaling pathway [23]. Moreover, VD can inhibit the ferroptosis of zebrafish liver cells by regulating the Kelch-like ECH-associated protein 1 (Keap1)-Nrf2-GPx4 and NF-κB-hepcidin axis [24]. However, the research on the relationship between VD and ferroptosis in the nervous system is limited. Nrf2 is a transcription factor that regulates the cellular antioxidant response and maintains redox homeostasis. The target gene of Nrf2 is involved in regulating lipid peroxidation and ferroptosis [25]. Additionally, studies have shown that leonurine protects against cisplatin-induced acute kidney injury by inhibiting ferroptosis through the Nrf2 signaling pathway [26]. Research has shown that dexmedetomidine can protect the heart against MIRI-induced ferroptosis by activating Nrf2 through the AMPK/GSK-3β signaling pathway [27]. Itaconate has been shown to inhibit ferroptosis in macrophages through the Keap1/Nrf2/HO-1 pathway, thereby protecting against sepsis-induced acute lung injury [28]. These studies suggest that the VDR and Nrf2 play important roles in regulating ferroptosis. VD deficiency is a common issue among older individuals. However, whether VD can inhibit ferroptosis and influence the development and progression of neurodegenerative diseases through the VDR/Nrf2 pathway is still unknown.

Therefore, this study aimed to investigate the effect of VD on learning and memory in aging mice and its potential to reduce damage to aging hippocampal neurons by inhibiting ferroptosis. We used C57BL/6 mice and HT22 cells to establish aging models by using D-gal, and the results show that ferroptosis played a critical role in aging. The results also show that VD could activate the Nrf2/HO-1 signaling pathway through its target receptor, the VDR, which inhibited ferroptosis and protected against hippocampal neuron damage caused by aging. These findings offer new insights into the mechanism underlying aging-related neurodegenerative diseases.

## 2. Results

### 2.1. VD Improves Learning and Memory in D-gal-aged Mice

The results of the open field and elevated plus maze tests showed that different administration methods did not affect emotions (Supplementary Figure S1).

**Figure 1:**
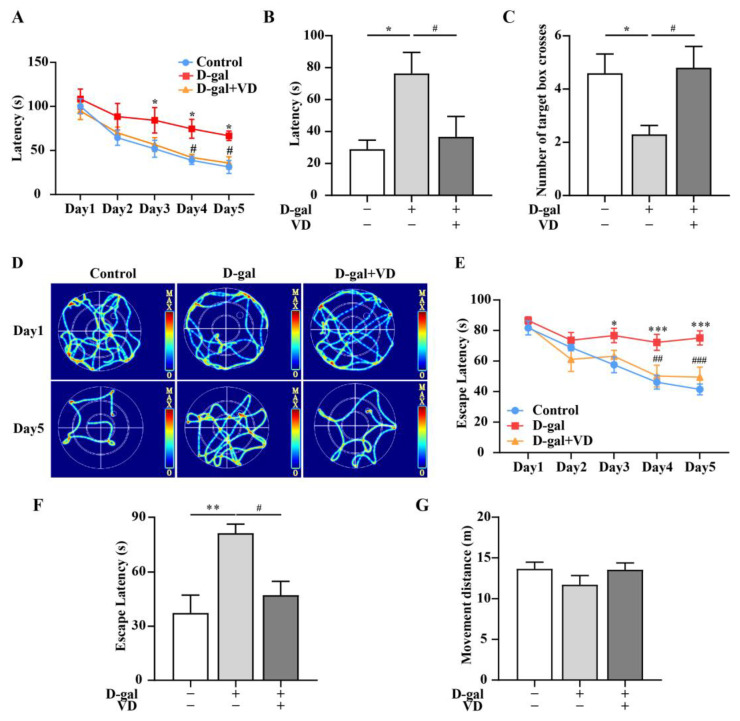
VD improves learning and memory in D-gal-aged mice. (**A**) Latency during training. (**B**) Latency on the sixth day. (**C**) The number of target box crosses on the sixth day. (**D**) Heat map of mouse movement trajectory. (**E**) Escape latency during training. (**F**) Escape latency on the sixth day. (**G**) Movement distance on the sixth day. (*n* = 10). Compared with the control group, * *p* < 0.05, ** *p* < 0.01, *** *p* < 0.001; compared with the D-gal group, # *p* < 0.05, ## *p* < 0.01, ### *p* < 0.001.

To assess spatial memory, it is recommended to use at least two different test systems: the land maze (Barnes maze) and the water maze (Morris water maze) [29]. In the Barnes maze experiment, the D-gal group exhibited a significantly higher latency than the control group. On the other hand, the D-gal+VD group demonstrated a significant reduction in latency compared to the D-gal group (Figure 1A). In the probe experiment, which was conducted on the sixth day, the latency was reduced in the D-gal+VD group compared to the D-gal group. Additionally, the D-gal+VD group exhibited an increase in the number of target box crossings (Figure 1B,C). The results of the Morris water maze experiment showed that during the training days, the D-gal group had a significantly higher escape latency than the control group. The movement track of control mice was clear, and they were able to find the escape platform quickly. On the other hand, the D-gal group exhibited aimless searching behavior in attempting to find the escape platform. However, the D-gal+VD group showed movement tracks similar to those of the control group (Figure 1D) and exhibited a significantly reduced escape latency compared to that of the D-gal group (Figure 1E). During the space exploration experiment, which was conducted on the sixth day, the D-gal group exhibited a significant increase in escape latency. In contrast, the D-gal+VD group exhibited a significantly reduced escape latency (Figure 1F). However, there were no notable differences in the movement distance of the mice in the different groups (Figure 1G).

### 2.2. Effects of VD on D-gal-Induced Hippocampal Damage and Ferroptosis in Mice

**Figure 2:**
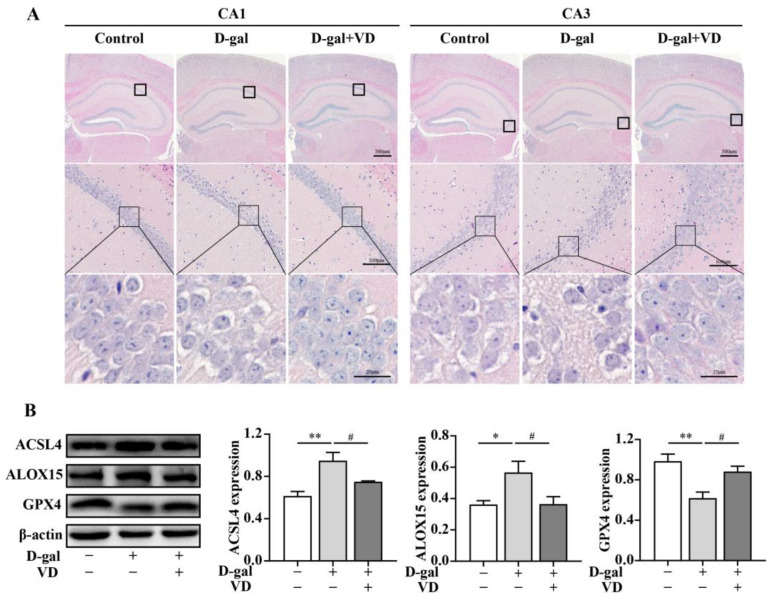
VD improves the structure of hippocampal neurons in D-gal-aged mice and inhibits ferroptosis. (**A**) Hippocampal morphology following HE staining (bar = 25 μm, 100 μm, 500 μm). (**B**) Protein expression of ACSL4, ALOX15, and GPX4 proteins in mouse hippocampal tissues (*n* = 5). Image J version 1.51j8 software was used to quantify the relative density of ASCL4, ALOX15, and GPX4 relative to β-actin. Compared with the control group, * *p* < 0.05, ** *p* < 0.01; compared with the D-gal group, # *p* < 0.05.

The hippocampus is crucial for learning and memory, particularly spatial navigation and the integration of information from short-term to long-term memory. The CA1 and CA3 regions of the hippocampus play essential roles in these processes [30,31]. To evaluate the effect of VD on hippocampal neuron structure and ferroptosis in D-gal-aged mice, we used HE staining and Western blotting. The results showed that in the control group, the number of hippocampal cells was increased; they had a close and regular arrangement, and the nucleus was apparent. In contrast, the hippocampal neurons in the CA1 and CA3 regions of the D-gal group exhibited a sparse and irregular arrangement, and there was a certain degree of neuron loss. The cell structure was unclear, and the nucleus was not prominent. However, these negative effects were reversed in the D-gal+VD group (Figure 2A). NeuN, a marker of mature neurons, is involved in regulating nerve cell differentiation and nervous system development. We examined the expression of NeuN in the hippocampus via Western blotting. A significant decrease in NeuN expression was observed in the D-gal group compared to the control group. However, in the D-gal+VD group, there was a significant increase in NeuN expression (Supplementary Figure S2). To further investigate the effect of VD on ferroptosis, we examined the expression levels of specific markers. The results showed that in the D-gal group, there was an increase in acyl-CoA synthetase long-chain family member 4 (ACSL4) and arachidonate 15-lipoxygenase (ALOX15), and GPX4 was downregulated. However, in the D-gal+VD group, these changes were significantly reversed (Figure 2B). This finding suggests that VD protects against ferroptosis by regulating the expression of these markers in the hippocampus of D-gal-aged mice.

**Figure 3:**
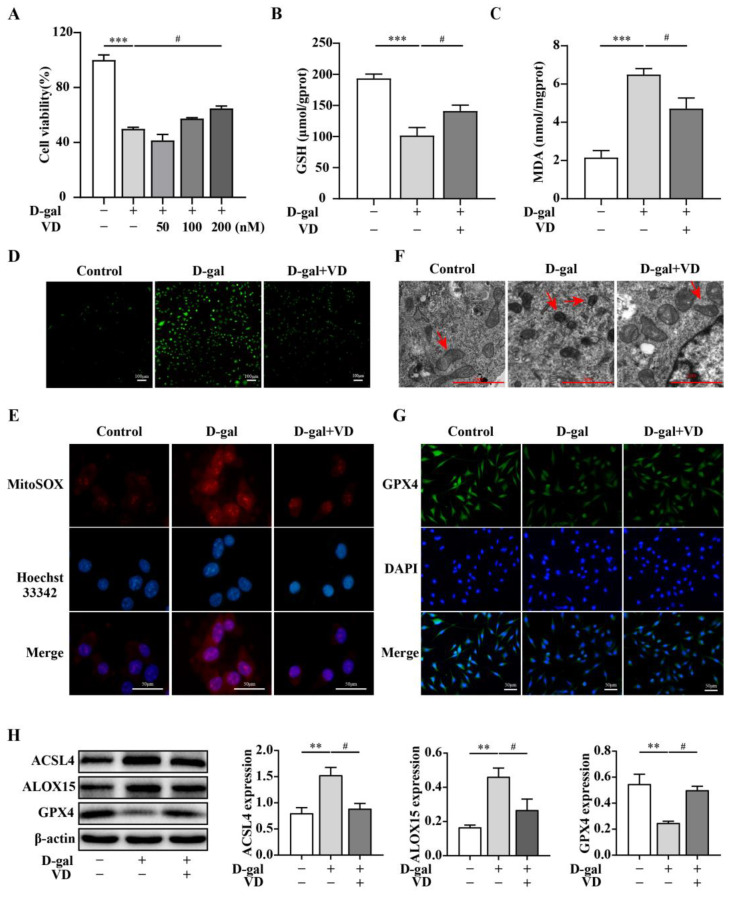
VD alleviates D-gal-induced ferroptosis in HT22 cells. The HT22 cells were treated with D-gal (250 mM) and VD (0–200 nM) for 24 h. (**A**) The CCK-8 method was used to detect cell viability (*n* = 3). (**B**,**C**) Intracellular GSH and MDA levels (*n* = 4). (**D**) DCFH-DA measured intracellular ROS generation. ROS was green in DCFH-DA fluorescent staining (bar = 100 μm). (**E**) Mitochondrial ROS levels determined via MitoSOX staining; mitochondrial ROS was red in MitoSOX staining (bar = 50 μm). (**F**) Transmission electron microscope observation of mitochondrial morphology. The red arrow indicates the mitochondria (bar = 2 μm). (**G**) Immunofluorescence to detect the expression of GPX4 (bar = 50 μm). (**H**) Protein expression of ACSL4, ALOX15, and GPX4 (*n* = 4). Image J software was used to quantify the relative density of ACSL4, ALOX15, and GPX4 relative to β-actin. Compared with the control group, ** *p* < 0.01, *** *p* < 0.001; compared with the D-gal group, # *p* < 0.05.

To investigate the protective effect of VD on hippocampal neurons in greater detail, HT22 cells were exposed to different concentrations of D-gal for 24 h. The results demonstrated that cell viability decreased by 50% when the concentration of D-gal reached 250 mM (Supplementary Figure S3A). Subsequently, HT22 cells were treated with 250 mM D-gal to induce cell aging. In response to treatment with different concentrations of VD, the increase in cell viability was dose dependent, and there was a notable effect observed in response to 200 nM VD treatment (Figure 3A). Subsequently, we examined the levels of intracellular lipid peroxidation and mitochondrial morphology. The results demonstrated that GSH levels decreased, while MDA and intracellular and mitochondrial ROS levels increased in the D-gal group. The fluorescence intensity of ROS also increased, and the mitochondria appeared smaller with reduced cristae structures. Additionally, total iron and Fe^2+^ levels also increased in the D-gal group. However, VD significantly reversed these changes, restoring the GSH levels, reducing MDA and intracellular and mitochondrial ROS levels and total iron and Fe^2+^ levels, and improving mitochondrial morphology (Figure 3B–F) (Supplementary Figure S3B–D). We examined the expression of ACSL4, ALOX15, and GPX4. The results showed that GPX4 expression was downregulated, while ACSL4 and ALOX15 expression was upregulated in the D-gal group. However, VD effectively reversed these changes, restoring GPX4 expression and reducing ACSL4 and ALOX15 expression levels (Figure 3G,H). These results highlight the potential of VD treatment to mitigate ferroptosis in the aging hippocampus.

### 2.3. VD Upregulates the Expression of the Nrf2 Signaling Pathway via the VDR

**Figure 4:**
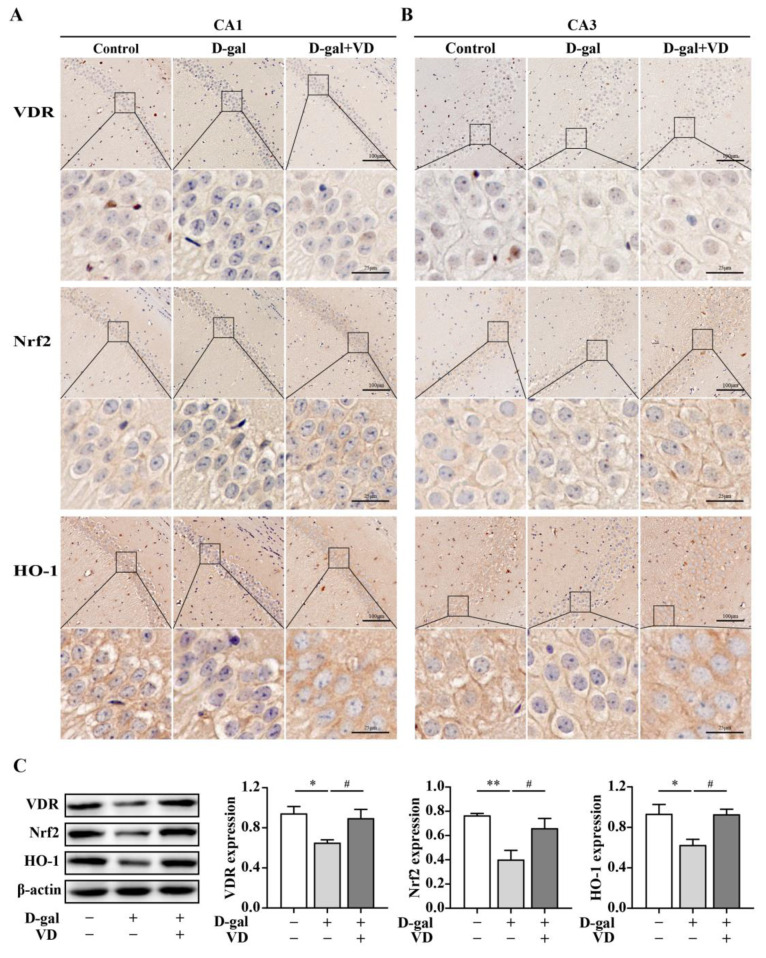
VDR/Nrf2/HO-1 expression in hippocampus. (**A**,**B**) Immunohistochemistry was used to detect the expression of the VDR, Nrf2, and HO-1 in the hippocampus in the CA1 and CA3 regions (bar = 25μm, 100 μm). (**C**) Protein expression of the VDR (*n* = 6), Nrf2, and HO-1 (*n* = 4) in the hippocampal regions. Image J software was used to quantify the VDR, Nrf2, and HO-1 relative density values relative to β-actin. Compared with the control group, * *p* < 0.05, ** *p* < 0.01; compared with the D-gal group, # *p* < 0.05.

To investigate the regulation of VDR, Nrf2, and HO-1 expression by VD, we used immunohistochemistry and Western blotting. The immunohistochemical results showed that VDR, Nrf2, and HO-1 expression levels in the hippocampal CA1 and CA3 regions in the D-gal group were downregulated compared with that in the control group. In contrast, in the D-gal+VD group, the expression levels of these proteins were increased. The VDR was mainly expressed in the nucleus in hippocampal neurons, while Nrf2 and HO-1 were mainly expressed in the cytoplasm in hippocampal neurons (Figure 4A,B). Additionally, the Western blot results confirmed that the VDR, Nrf2, and HO-1 expression levels were significantly downregulated in the D-gal group compared with the control group. Conversely, in the D-gal+VD group, these changes were reversed, restoring the expression levels of the VDR, Nrf2, and HO-1 (Figure 4C).

**Figure 5:**
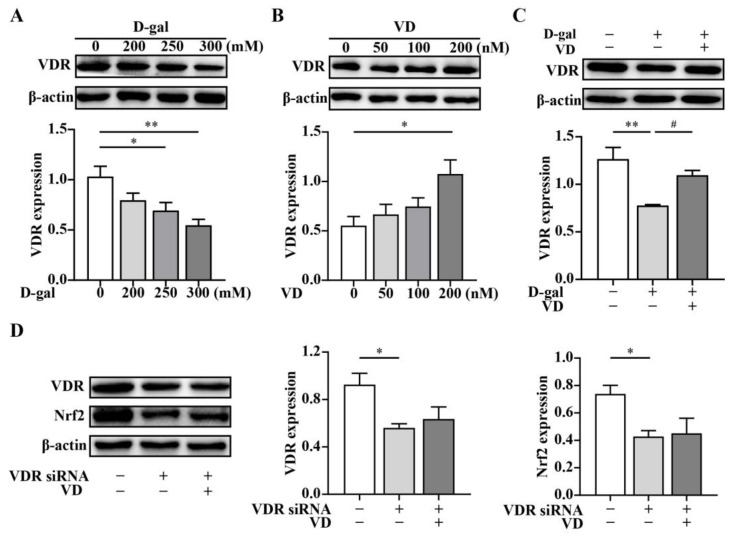
VD regulates the expression of Nrf2 via VDR. The HT22 cells were treated with D-gal (0–300 mM) and VD (0–200 nM) for 24 h. (**A**–**C**) Protein expression of the VDR (*n* = 4). The HT22 cells were treated with VDR siRNA for 24 h and subsequently treated with VD (200 nM) for 24 h. (**D**) Protein expression of the VDR and Nrf2 (*n* = 4). Image J software was used to quantify VDR and Nrf2 relative density values relative to β-actin. Compared with the control group, * *p* < 0.05, ** *p* < 0.01; compared with D-gal group, # *p* < 0.05.

To further investigate whether VD regulates the expression of Nrf2 through the VDR, we examined the effects of various concentrations of D-gal and VD on VDR expression. The results showed that VDR protein expression decreased in a dose-dependent manner when HT22 cells were cultured with increasing concentrations of D-gal compared to untreated cells (Figure 5A). Conversely, the expression of the VDR dose-dependently increased with increasing concentrations of VD (Figure 5B). Moreover, VDR expression was upregulated after VD treatment compared with that in the D-gal group (Figure 5C). To determine the specificity of VD-induced Nrf2 in HT22 cells, we performed VDR siRNA silencing to knock down VDR expression. Our results indicated that VDR expression was significantly reduced in the cells transfected with VDR siRNA compared to the control cells. VD did not upregulate VDR expression when the cells were treated with VDR siRNA. Notably, Nrf2 expression was markedly downregulated in the cells that were transfected with VDR siRNA, and VD could not upregulate Nrf2 expression when VDR was knocked down in HT22 cells (Figure 5D). These findings suggest that VD can regulate the expression of Nrf2 through the VDR in HT22 cells, highlighting the involvement of the VDR/Nrf2 pathway in the neuroprotective effects of VD.

### 2.4. VD Inhibits Ferroptosis by Upregulating the Expression of the Nrf2 Signaling Pathway

**Figure 6:**
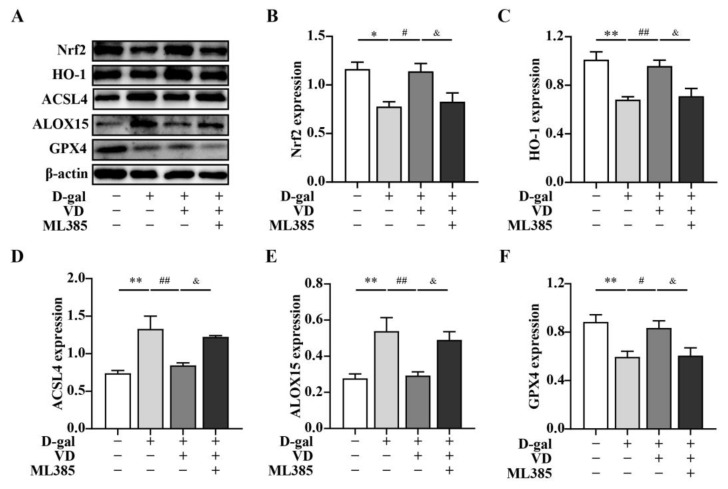
VD inhibits ferroptosis through the Nrf2/HO-1 pathway. The HT22 cells were treated with D-gal (250 mM) and VD (200 nM) for 24 h. Prior to this treatment, the HT22 cells were pretreated with ML385 for 1 h. (**A**–**F**) Protein expression of Nrf2, HO-1, ACSL4, ALOX15 (*n* = 5), GPX4 (*n* = 7). Image J software was used to quantify Nrf2, HO-1, ACSL4, ALOX15, and GPX4 relative density values relative to β-actin. Compared with the control group, * *p* < 0.05, ** *p* < 0.01; compared with the D-gal group, # *p* < 0.05, ## *p* < 0.01; compared with the D-gal + VD group, & *p <* 0.05.

In this study, we aimed to investigate whether VD modulated ferroptosis via the Nrf2/HO-1 pathway. To examine this, HT22 cells were treated with D-gal and VD. The results demonstrated that when HT22 cells were treated with D-gal and VD, the expressions levels of Nrf2 and HO-1 were increased compared with those in the D-gal group. This finding suggests that VD treatment can enhance the activation of the Nrf2/HO-1 pathway. To further elucidate the role of the Nrf2/HO-1 pathway in the VD-mediated effects, we treated the cells with the Nrf2 inhibitor ML385. Compared with the effect of D-gal and VD, the expression levels of Nrf2 and HO-1 were significantly downregulated in the presence of ML385. Additionally, the expression levels of ACSL4 and ALOX15, which are markers of ferroptosis, were increased, and the expression of GPX4 was reduced (Figure 6). These findings suggest that VD can modulate ferroptosis through the Nrf2/HO-1 pathway, thereby contributing to its neuroprotective effects.

Overall, our results indicate that VD treatment can enhance Nrf2 and HO-1 expression, leading to the modulation of ferroptosis in HT22 cells. This finding suggests the role of the Nrf2/HO-1 pathway in the neuroprotective effects of VD.

## 3. Discussion

Aging is a significant risk factor for the majority of neurodegenerative diseases [32]. D-gal is commonly used to induce aging in model animals, as it accelerates the aging process by promoting the formation of advanced glycation end products (AGEs) through nonenzymatic glycosylation reactions [33]. Numerous studies have indicated that D-gal exposure can lead to hippocampal neuron damage and impair learning and memory in C57BL/6J mice [5,34].

VD is a necessary fat-soluble vitamin that plays a crucial role in calcium and phosphorus metabolism, bone development, and cell differentiation and proliferation [35]. Insufficient levels of VD have been associated with various health conditions, such as osteoporosis, heart disease, hypertension, autoimmune disorders, cancer, and stroke [36]. Previous research by our team demonstrated that VD improved the inflammatory response of the placenta, reduced the risk of preeclampsia (PE), and regulated the miR26b-5p-COX2 pathway through the VDR [37]. Numerous studies have shown that the administration of VD can enhance learning and memory in the progeny of preeclampsia rats. Conversely, VD deficiency has been linked to learning and memory impairment in mice [38,39]. In this study, the aging model was established by D-gal. Previous studies have shown that D-gal can lead to cognitive decline and injury to hippocampal neurons in mice [40,41]. We used the Morris water maze and Barnes maze to evaluate the impact of VD on learning and memory in D-gal-aged mice. The results demonstrated that VD treatment decreased the time taken to locate the target by the D-gal-aged mice, suggesting that VD enhanced their learning and memory. It is worth noting that the function of hippocampal neurons is associated with learning and memory capacity [42,43]. Subsequently, we examined the structure of hippocampal neurons. The results indicated that VD treatment had a positive effect on the structure of hippocampal neurons in D-gal-aged mice. NeuN is a specific nuclear protein found in neurons and is commonly used as a marker of neurogenesis. Consistent with previous results, we observed a significant reduction in the expression of NeuN in the D-gal group. However, the administration of VD could increase the expression of NeuN in the D-gal group, suggesting that VD treatment improved the damage caused by D-gal in the hippocampus.

Ferroptosis is a form of cell death that relies on iron and is increasingly recognized as playing a role in various neurological diseases, such as stroke, Alzheimer’s disease, Parkinson’s disease, and traumatic brain injury [9]. GPX4 is a crucial regulator of ferroptosis that functions as a critical enzyme that reduces phospholipid hydroperoxides. Inhibiting GPX4 activity leads to the accumulation of lipid peroxides [44]. Polyunsaturated fatty acids (PUFAs) are important targets of lipid peroxidation, and they play a crucial role in ferroptosis. Proteins such as ACSL4 and lysophosphatidylcholine acyltransferase 3 (LPCAT3) facilitate the esterification of free PUFAs such as arachidonic acid and adrenergic acid into phosphatidylethanolamine (PE). Subsequently, lipoxygenases such as ALOX15 catalyze the conversion of PE into reactive intermediates that induce cellular ferroptosis [45,46]. To investigate whether the protective effect of VD on hippocampal neurons in D-gal-aged mice involves ferroptosis, we examined the expression levels of GPX4, ACSL4, and ALOX15. The in vitro and in vivo results demonstrated that D-gal could induce ferroptosis in mouse hippocampal neurons. However, VD treatment could reverse the changes in ferroptosis-related protein expression caused by D-gal, such as the upregulation of ACSL4 and ALOX15 and downregulation of GPX4. ACSL4 and ALOX15 are critical for ferroptosis, and inhibiting their activity was shown to reduce ferroptosis induced by arachidonic acid (AA) and iron [47].

Ferroptosis is characterized by increased levels of lipid peroxidation, decreased mitochondrial volume, and reduced levels of GSH, in addition to the protein changes mentioned earlier [48]. Studies have shown that ferroptosis serves as a mechanism in statin-induced myopathy, and ferroptosis inhibitors could reduce elevated intracellular iron levels, intracellular and mitochondrial ROS levels, and lipid peroxidation induced by atorvastatin [49]. In our in vitro experimental model, we found that D-gal increased intracellular and mitochondrial ROS, MDA, and total iron and Fe^2+^ levels and reduced GSH levels. Furthermore, D-gal reduced mitochondrial volume, increased double membrane density, and reduced the disappearance of the mitochondrial ridge. However, treatment with VD could reverse these changes. Previous research by Xu et al. showed that VD could inhibit lipid peroxidation and restore mitochondrial morphology [23]. In conclusion, VD treatment may reduce the damage caused by D-gal to hippocampal neurons by regulating ferroptosis.

Subsequently, we conducted further investigations to determine whether VD has an impact on the Nrf2/HO-1 signaling pathway through the VDR to protect hippocampal neurons. The in vitro and in vivo experiments showed that VD could upregulate the aging-related decline in the protein expression of VDR/Nrf2/HO-1. These findings suggest that VD protects aging hippocampal neurons by engaging with the VDR to regulate the Nrf2/HO-1 pathway.

To further elucidate the regulatory role of the VDR, we used siRNA to knockdown the expression of the VDR in HT22 cells and examined changes in Nrf2 expression. Our findings revealed that VD treatment was unable to upregulate Nrf2 expression following VDR knockdown, suggesting that VD regulates Nrf2 expression via VDR. Additionally, the activation of the VDR/Nrf2/HO-1 pathway by VD has been shown to inhibit the activation of the NLRP1 inflammasome in unprimed keratinocytes in response to nigericin [50]. Yang et al. demonstrated that silencing VDR expression in BEAS-2B cells inhibited the Nrf2 signaling pathway [51]. Moreover, Li et al. found a positive association between Nrf2 and VDR expression levels, and in their study, eriodictyol activated the Nrf2/HO-1 signaling pathway through the VDR to mitigate the pathological effects of Alzheimer’s disease [52]. These studies collectively indicate that VD activates the Nrf2 signaling pathway through VDR.

Nrf2 functions as a critical regulatory factor in the antioxidant response; after translocating to the nucleus, it activates the expression of downstream genes, including HO-1, thereby enhancing the antioxidant response [53]. Nrf2 plays a vital role in the onset, progression, and treatment of neurodegenerative disorders such as Alzheimer’s disease, Parkinson’s disease, and Huntington’s disease [54,55,56]. Furthermore, Nrf2 serves as a crucial modulator of lipid peroxidation and ferroptosis [25]. Many drugs inhibit ferroptosis by regulating the Nrf2/HO-1 signaling pathway. For instance, the activation of Nrf2/HO-1 by ferrostatin-1 has been shown to protect against liver injury induced by diabetes [57]. Panaxydol has been shown to alleviate ferroptosis in LPS-induced acute lung injury through the Keap1-Nrf2/HO-1 pathway [58]. Additionally, gastrodin has been shown to protect HT-22 cells against glutamate-induced ferroptosis via the Nrf2/HO-1 signaling pathway [59]. We further examined whether VD modulates ferroptosis through the Nrf2/HO-1 pathway. The Nrf2 inhibitor ML385 was used to suppress Nrf2 expression, which resulted in significant decreases in Nrf2 and its downstream gene HO-1. Moreover, while the expression level of GPX4 decreased, the expression of the ferroptosis-related indicators ACSL4 and ALOX15 increased. These findings indicated that VD could inhibit ferroptosis by upregulating the Nrf2 signaling pathway.

**Figure 7:**
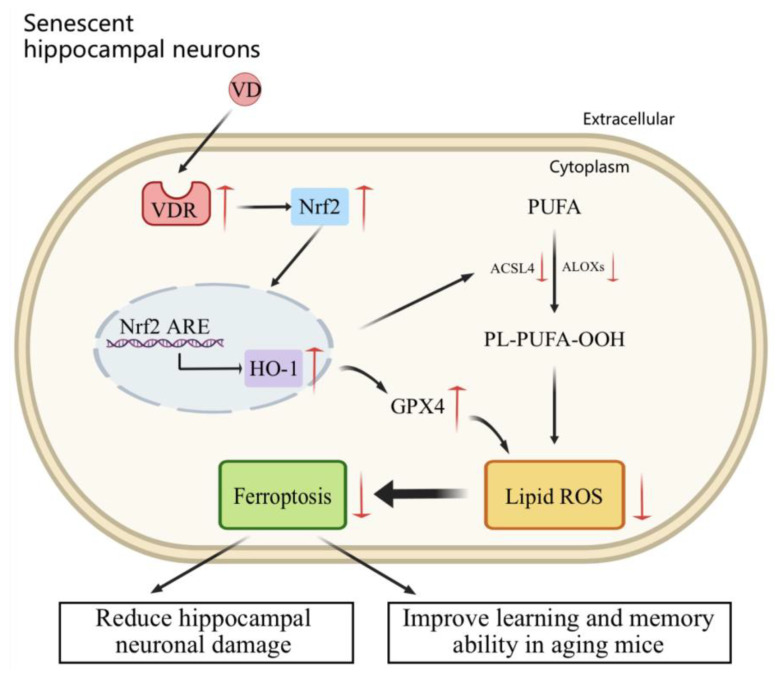
The anti-aging mechanism of VD in aging hippocampal neurons. VD binds to its receptor, the VDR, which inhibits ferroptosis by upregulating the Nrf2/HO-1 pathway, reduces hippocampal neuronal damage, and improves learning and memory in aging mice.

In conclusion, this study elucidated the mechanism by which VD acts on hippocampal neurons in D-gal-aged mice. VD activates the Nrf2/HO-1 signaling pathway through its interaction with the VDR, thereby reducing ferroptosis and enhancing learning and memory in aging mice (Figure 7). These findings suggest that VD may play a role in the prevention and adjunctive treatment of aging-related neurodegenerative diseases. Furthermore, these results provide valuable insights into the clinical application of VD to treat related diseases, which has important guiding significance.

## 4. Materials and Methods

### 4.1. Animals and Experimental Models

Thirty male C57BL/6J mice (3-month-old) were provided by the Second Affiliated Hospital of Harbin Medical University. The mice were housed in a controlled environment with natural conditions, including a suitable indoor temperature and humidity, compliance with natural light cycles, and ad libitum access to water. All experimental procedures were approved by the Institutional Animal Care and Use Committee (IACUC) of Harbin Medical University.

The mice were randomly divided into three groups, with each group containing 10 mice:

Control (0.9% NaCl: ip, every day/sc every other day);
D-gal group (D-gal: 150 mg/kg, ip, every day) [60];
D-gal+VD group (D-gal: 150 mg/kg, ip, every day; VD: 1 μg/kg, sc, every other day) [61].
D-gal (Macklin, Shanghai, China), 1,25(OH)_2_D_3_ (TargetMo, Shanghai, China) is a bioactive VD. The doses were administered to the mice for eight weeks. All drugs were dissolved in 0.9% NaCl. In this study, different behavioral tests were conducted to evaluate the effects of drug administration on mice. During the sixth week of drug administration, open field and elevated plus maze tests were used to assess the emotions in the mice. Subsequently, during the seventh and eighth weeks, the Barnes Maze and Morris Water Maze tests were used to evaluate the learning and memory abilities of the mice. These tests provided valuable information on the impact of drug administration on various behavioral aspects, including emotions, as well as learning and memory capabilities.

### 4.2. Behavioral Experiments

#### 4.2.1. Barnes Maze

The Barnes maze is a dry land maze that is commonly used to assess visual–spatial learning and memory in research studies. It consists of a circular platform with a diameter of 75 cm and a height of 50 cm. Around the periphery of the platform, there are eighteen holes, and one of them leads to a box. To facilitate spatial navigation, visual cues were placed around the experimental room to help the mice identify the location of the platform. Each mouse was given a maximum exploration time of 3 min. If the mouse failed to reach the target within this time, it was gently removed from the maze and guided to the target box, where it remained for 15 s. The test was conducted over five consecutive days, with three trials per day and intervals of 15–20 min. The time taken by each mouse to find the target box was recorded during each trial. A spatial probe test was performed on the sixth day. The target box was blocked, and the mice were allowed to freely explore the maze for 2 min. During this probe test, the latency (time taken to reach the target) and the number of times the mice traverse the target box were recorded.

#### 4.2.2. The Water Maze

The Morris water maze test was used to assess the learning and memory of the mice in each group. The water maze apparatus consisted of a circular water tank measuring 120 cm in diameter and 50 cm in height. The water tank was divided into four equal quadrants. In this test, a circular escape platform measuring 6.5 cm in diameter and 14 cm in height was positioned 1 cm below the water surface in the middle of one fixed quadrant. The mice were randomly placed in one of the quadrants and allowed to swim until they found the platform or until 90 s had elapsed. If a mouse failed to locate the platform within the allotted time, it was gently guided to the platform and allowed to spend 15 s there. The Morris water maze test was conducted for five consecutive days, with each mouse undergoing four training trials per day, each lasting 90 s. The time taken by the mice to find the escape platform was recorded during the training trials. On the sixth day, a spatial probe test was performed. During this test, the escape platform was removed, and the mice were given 90 s to freely swim in the maze. The latency (time taken to reach the previous location of the escape platform) and the distance traveled were recorded and analyzed.

#### 4.2.3. Elevated plus Maze Test

Anxiety-like behavior was assessed using the elevated plus maze test, which involved a maze with dimensions of 60 cm length, 5 cm width, and 50 cm height. The test involved placing each mouse in the central area of the maze, facing one of the open arms. The mice were allowed to explore the maze freely for 5 min. During the test, several parameters were recorded to evaluate anxiety-like behavior, including the number of times the mouse entered the open arm, the percentage of the staying time, and the percentage of the movement distance in the open arm.

#### 4.2.4. Open Field Test

Anxiety-like behavior was assessed using an open field apparatus that was 50 cm in length, 50 cm in width, and 40 cm in height. Each mouse was placed in the center of the open field apparatus and allowed to freely explore for 5 min. Several parameters were recorded, including the distance traveled by the mouse, average speed, rest time, and distance traveled in the central region.

### 4.3. Hematoxylin and Eosin (HE) Staining

Mice were first perfused with PBS and then with 4% formaldehyde solution. After isolation brain, the tissues were fixed in 4% formaldehyde for 48 h, followed by dehydration using a 30% sucrose solution for 3 h. The next step involved paraffin embedding and sectioning using the rotator microtome, which cut the tissue into serial sections that were 5 μm thick. The sections were dewaxed with xylene, rehydrated with graded concentrations of ethanol, and stained with hematoxylin and eosin.

### 4.4. Immunohistochemistry

Paraffin sections of brain tissue were first dewaxed using xylene and then rehydrated by being graded with a series of ethanol solutions of increasing concentration. Next, the sections were incubated in citrate buffer (pH 6.0) at 95 °C for antigen retrieval and subsequently cooled for 1 h at room temperature. After treating the sections with 3% hydrogen peroxide (H_2_O_2_) for 10 min in a dark room to block endogenous peroxidase activity, they were blocked for 1 h. Following that, the sections were incubated overnight at 4 °C with the primary antibodies of the following: VDR (1:100) (Affinity, Changzhou, China), Nrf2 (1:100), and HO-1 (1:50) (Abcam, Cambridge, UK). Subsequently, the sections were incubated with the secondary antibody at 37 °C for 1 h and room temperature for 1 h. To visualize the target proteins, the sections were then counterstained with DAB and hematoxylin.

### 4.5. Western Blot Analysis

The protein concentration in the hippocampus or cells was detected using a BCA kit (Meilunbio, Dalian, China). Briefly, the total protein was separated by 10% SDS-PAGE and transferred to a polyvinylidene difluoride (PVDF) membrane. Following the blocking step with 5% nonfat milk for 2 h at room temperature, the membranes were incubated overnight at 4 °C with specific primary antibodies, namely β-actin (1:2000) (Elabscience Biotechnology, Wuhan, China), ALOX15 (1:500) (Santa Cruz Biotechnology, Santa Cruz, CA, USA), Nrf2 (1:500), HO-1 (1:500), VDR (1:1000), GPX4 (1:1000), NeuN (1:1000), and ACSL4 (1:500) (Affinity, Changzhou, China). After washing by TBST, the membranes were incubated with peroxidase-conjugated goat anti-rabat or goat anti-mouse IgG (EarthOx, San Francisco, CA, USA) for 1 h at room temperature. Then, the protein bands were visualized using Pierce ECL Western blotting Substrate (Engreen Biosystem, Beijing, China). The relative optical density values of protein bands were quantified using Image J software (National Institutes of Health, Bethesda, MD, USA).

### 4.6. Cell Culture and Treatment

Mouse hippocampus-derived neuronal HT22 cells (Merck KGaA, Darmstadt, Germany) were cultured in MEM medium containing 10% fetal bovine serum and 1% penicillin/streptomycin and incubated under standard conditions of 5% CO_2_ at 37 °C. The cells were sub-cultured or treated with drugs when they reached approximately 80% confluence. In the experiment, the HT22 cells were treated with D-gal at a concentration of 250 mM and 1,25(OH)_2_D_3_ at a concentration of 200 nM 24 h. Prior to treatment, the HT22 cells were pretreated with 20 μM ML385 (MedChemExpress, Piscataway, NJ, USA) for 1 h. At the end of each experiment, the total cellular protein was extracted and used to determine protein expression.

### 4.7. CCK-8 Assay

The HT22 cells were seeded at a density of 4 × 10^3^ cells per well in 96-well plates and incubated overnight for 24 h. After being treated with D-gal or D-gal and VD for 24 h, the cells were treated with CCK-8 reagent (Yiyuan Biotechnology, Guangzhou, China) and incubated for 2 h at 37 °C. The absolute optical density value was measured at a wavelength of 450 nm using a microplate reader.

### 4.8. VDR siRNA Transfection

To achieve VDR downregulation in the HT22 cells, VDR siRNA was transfected into the cells using the Lipofectamine 2000 transfection reagent (Invitrogen, Carlsbad, CA, USA) according to the manufacturer’s instructions. Briefly, the HT22 cells were incubated with Opti-MEM medium for 6 h, which contains 200 nM of VDR siRNAs (GenePharma, Suzhou, China) mixed with Lipofectamine transfection reagent. The cellular protein was collected 48 h after transfection, and the protein expression levels for the VDR and Nrf2 were then determined via Western blot.

### 4.9. GSH, MDA, and Total Iron Level Kit Assay

The HT22 cells were seeded at a density of 11 × 10^4^ cells per well in 6-well plates. After being treated with D-gal or D-gal and VD for 24 h, the cells were collected, and the supernatant was prepared according to the instructions. MDA, GSH, and total iron levels were measured using commercial biochemical kits (Jiancheng Bioengineering Institute, Nanjing, China) according to the manufacturer’s instructions.

### 4.10. Intracellular Fe^2+^ Level

The HT22 cells were seeded in 24-well plates at a density of 2.5 × 10^4^ cells per well. After being treated with D-gal or D-gal and VD for 24 h, the cells were cultured with 1 μM FerroOrange(Dojindo, Kumamoto, Japan) at 37 °C for 30 min. Additionally, cell nuclei were stained with Hoechst 33342 (Beyotime Biotechnology, Shanghai, China) for 5 min at room temperature. The fluorescence was observed under a fluorescence microscope and captured in photographs.

### 4.11. Intracellular ROS Assay

The HT22 cells were seeded in 96-well plates at a density of 4 × 10^3^ cells per well. The 2,7-dichlorodihydrofluorescence indiacetate (DCFH-DA) ROS probe (Beyotime Biotechnology, Shanghai, China) was used to detect intracellular ROS. The cells were incubated with 20 μM DCFH-DA probe at 37 °C for 30 min in the dark and then washed with PBS three times. The stained cells were reviewed under a microscope, and images were captured via the use of a digital scanning microscopy imaging system (PreciPoint, Freising, Germany).

### 4.12. Mitochondrial ROS Assay

The HT22 cells were seeded in 24-well plates at a density of 2.5 × 10^4^ cells per well. Mitochondrial ROS levels were measured using MitoSOX Red (Yeasen, Shanghai, China). Briefly, the HT22 cells were washed with Hank’s balanced salt solution (HBSS) and incubated in the dark with 5 μM MitoSOX Red for 15 min at 37 °C. Additionally, cell nuclei were stained with Hoechst 33342 for 5 min at room temperature. The stained cells were reviewed under a microscope, and images were captured by using a digital scanning microscopy imaging system.

### 4.13. Transmission Electron Microscopy

The HT22 cells were digested with trypsin and centrifuged at 3500 rpm for 10 min, and the supernatant was discarded, and glutaraldehyde was added and fixed overnight at 4 °C. After dehydration, immersion, embedding, patching, sectioning, and staining, the mitochondrial structure was observed via transmission electron microscopy (TEM).

### 4.14. Immunofluorescent Staining

The HT22 cells were seeded in 24-well plates at a density of 2.5 × 10^4^ cells per well and fixed with ice-cold 4% paraformaldehyde and permeabilized with 0.1% Triton X-100. Then, they were incubated with rabbit polyclonal antibody GPX4 (1:250) overnight at 4 °C. After that, the cells were incubated with Fluor488-conjugated goat anti-rabbit IgG (Yeasen, Shanghai, China) for 1 h at room temperature. Then, the cells were incubated with DAPI (Yeasen, Shanghai, China) and reviewed under a fluorescent microscope (Nikon Corporation, Tokyo, Japan).

### 4.15. Statistical Analysis

All data are presented as mean ± SEM. Group differences in the escape latency (Day 1–5) during the Morris water maze and Barnes maze test were analyzed using a two-way analysis of variance (ANOVA). Tukey’s multiple comparisons tests was then applied as a post hoc test to determine specific group differences. Another statistical analysis was performed using one-way ANOVA. GraphPad Prism 8 version 8.0.1.244 software was used to conduct these analyses. A value of *p* < 0.05 was considered statistically significant.

## Data Availability

Not applicable.

## Supplementary Materials

The supporting information can be downloaded at: https://www.mdpi.com/article/10.3390/ijms242015315/s1.
