# Supplementary material for: Vitamin D Improves Cognitive Impairment and Alleviates Ferroptosis via the Nrf2 Signaling Pathway in Aging Mice
Source: Int J Mol Sci. 2023 Oct 18;24(20):15315. doi: 10.3390/ijms242015315 (PMC10607218; doi:10.3390/ijms242015315)
Supplement: Supplementary file 1 [file ijms-24-15315-s001.zip › ijms-2625499-supplementary.pdf]

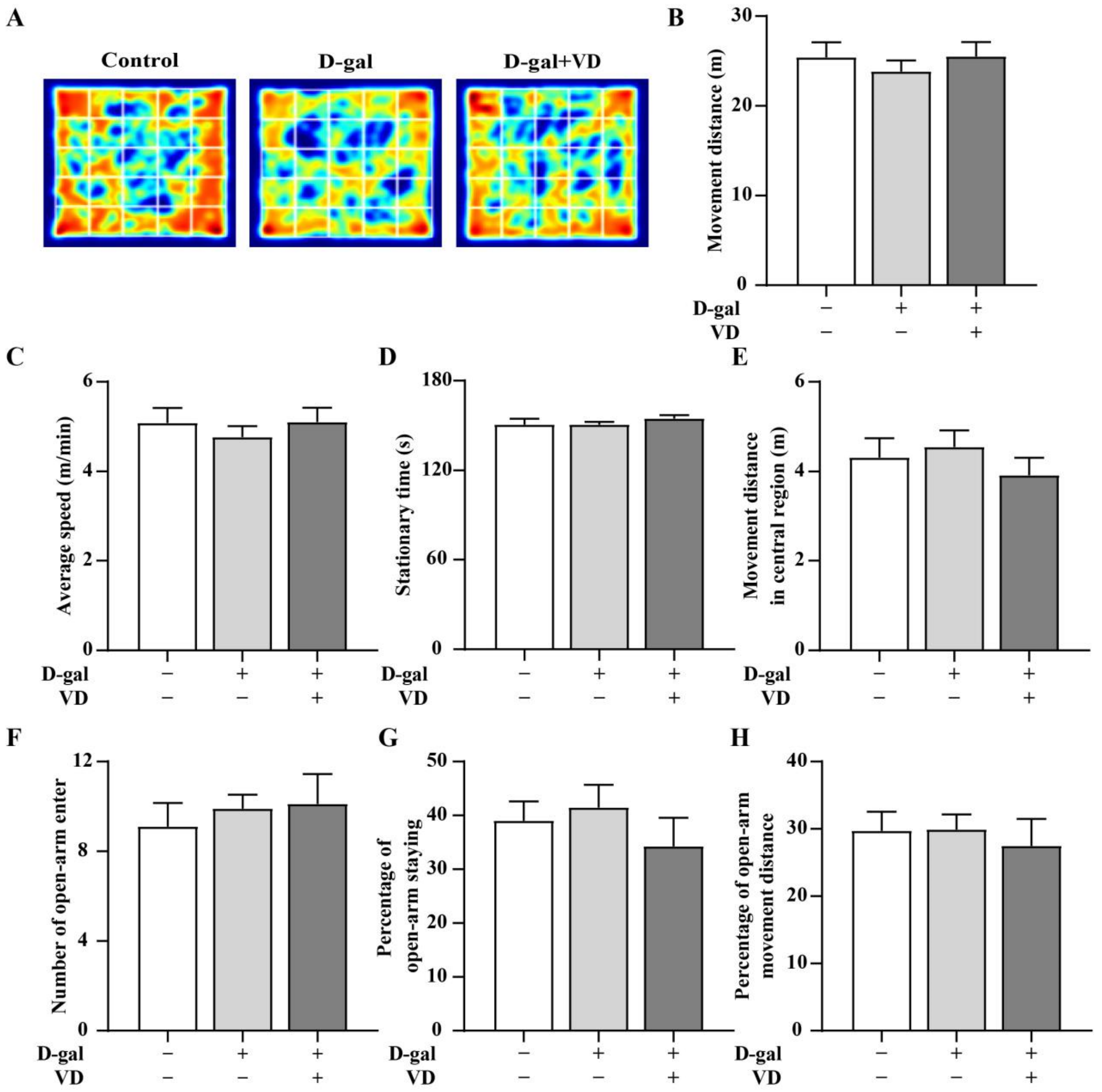

Supplementary Figure S1. No significant changes in mouse emotions. (A) Heat map of mouse movement trajectory in open field. (B) Movement distance in open field. (C) Average speed in open field. (D) Stationary time in open field. (E) Movement distance in the central area in open field. (F) The number of open-arm enter in elevated plus maze tests. (G) Percentage of open-arm staying in elevated plus maze tests. (H) The percentage of open-arm movement distance in elevated maze tests (*n*=10).

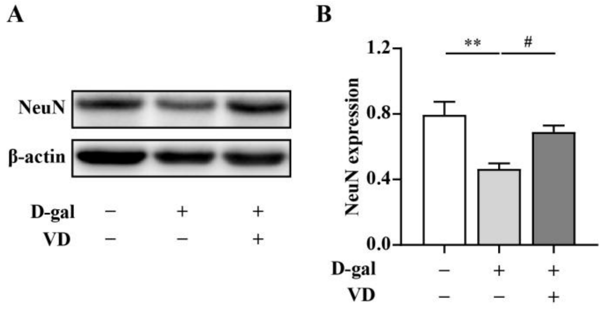

Supplementary Figure S2. NeuN expression in hippocampal. (A,B) Protein expression of NeuN (n=4). Image J software was use to quantify the relative density of NeuN relative to β-actin. Compared with the control group, **p<0.01; compared with the D-gal group, #p<0.05.

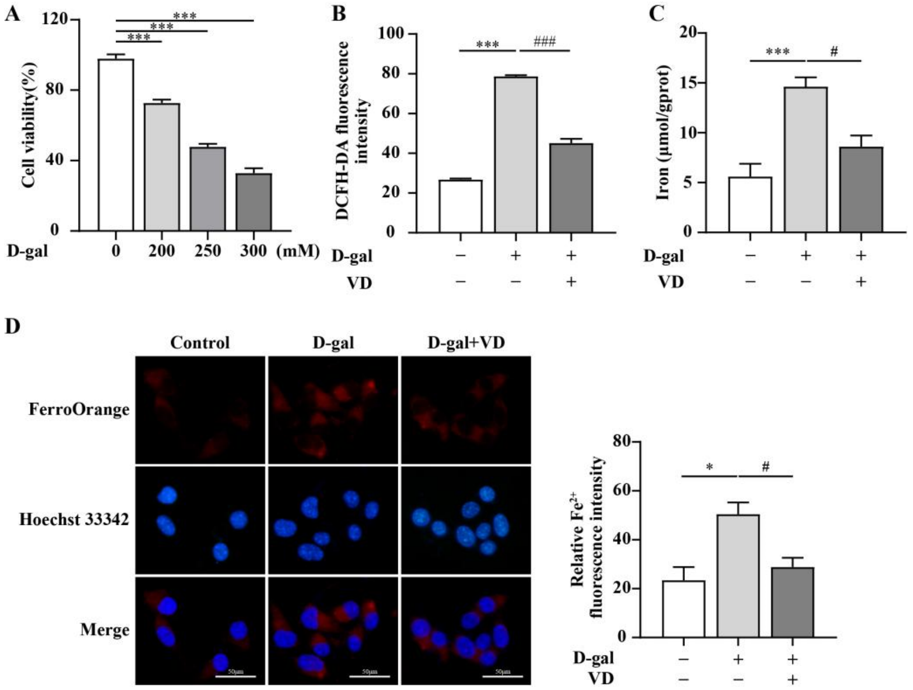

Supplementary Figure S3. Different concentrations of D-gal effects on HT22 cell viability, average fluorescence intensity and HT22 cell iron levels. HT22 cells were treated with D-gal (0-300 mM) for 24 h. (A) Different concentrations of D-gal effects on HT22 cell viability (n=5). (B) Average fluorescence intensity of DCFH-DA staining (n=3). (C) HT22 intracellular total iron levels. (D) HT22 intracellular Fe^{2+} was detected with the FerroOrange probe, bar = 50 μm. Fe^{2+} fluorescence intensity was quantified by ImageJ. Compared with the control group, *p<0.05,***p<0.001; compared with the D-gal group, #p<0.05, ###p<0.001.
